# Supplementary material for: Risk Assessment Considering the Bioavailability of 3-β-d-Glucosides of Deoxynivalenol and Nivalenol through Food Intake in Korea
Source: Toxins (Basel). 2023 Jul 18;15(7):460. doi: 10.3390/toxins15070460 (PMC10467052; doi:10.3390/toxins15070460)
Supplement: Supplementary file 1 [file toxins-15-00460-s001.zip › toxins-2448079-supplementary.pdf]

**Table S1.** Distribution of parameters using Monte Carlo simulation.

| **Parameter** | | | **Unit** | **Distribution (fitted parameters)** |
|---|---|---|---|---|
| Occurrence | DON group | Raw | μg/kg | Weibull |
| | | Simple | μg/kg | Log-normal |
| | | Fermented | μg/kg | Log-normal |
| | | Total | μg/kg | Log-normal |
| | NIV group | Raw | μg/kg | Gamma, Log-normal |
| | | Simple | μg/kg | Student's *t*, Logistic |
| | | Fermented | μg/kg | Log-normal |
| | | Total | μg/kg | Log-normal |
| Consumption | Raw | Infant | g/day | Log-normal |
| | | Adult | g/day | Log-normal |
| | | All ages | g/day | Log-normal |
| | Simple | Infant | g/day | Log-normal |
| | | Adult | g/day | Log-normal |
| | | All ages | g/day | Log-normal |
| | Fermented | Infant | g/day | Log-normal |
| | | Adult | g/day | Log-normal |
| | | All ages | g/day | Log-normal |
| | Total | Infant | g/day | Log-normal |
| | | Adult | g/day | Log-normal |
| | | All ages | g/day | Log-normal |
| bw | Infant | | kg | Log-normal |
| | Adult | | kg | Gamma |
| | All ages | | kg | Log-normal |
| TDI | DON group | | μg/kg bw/day | Fixed value 1.0 [7] |
| | NIV group | | μg/kg bw/day | Fixed value 0.4 (this study) |

DON, deoxynivalenol; NIV, nivalenol; bw; body weight TDI, tolerable daily intake.

**Table S2.** Summary of main toxicological studies for immunotoxicity and hemotoxicity of nivalenol (NIV).

| Species/sex | N | Duration | Dose | Endpoint | LOAEL/NOAEL | Reference |
|---|---|---|---|---|---|---|
| C57BL/6CrSlc SPF mice /female | 2–5 | 6 months | 0, 6, 12, 30 mg NIV/kg diet | Decrease in white blood cells | 30/12 | [30] [31] |
| | 6 | 1 year | | Decrease in white blood cells | -/- | |
| | 10 | 2 years | | Decrease in white blood cells | -/- | |
| C57B16 mice /male | 10 | 4 weeks | 0, 0.014, 0.071, 0.355, 1.774, 8.87 mg NIV/kg bw/3 times per week | Increase in serum IgG | 8.87/1.774 | [32] |
| F344/DuCrj rats /female | 9–10 | 90 days | 0, 6.25, 25, 100 mg NIV/kg diet | Decrease in white blood cells | 6.25/- | [33] [34] [35] |
| | | | | Decrease in red blood cells | -/- | |
| | | | | Decrease in platelets | 100/25 | |
| | | | | Decrease in hemoglobin | 100/25 | |
| F344/DuCrj rats /male | 9–10 | 90 days | 0, 6.25, 25, 100 mg NIV/kg diet | Decrease in white blood cells | 100/25 | |
| | | | | Decrease in red blood cells | 100/25 | |
| | | | | Decrease in platelets | 100/25 | |
| | | | | Decrease in hemoglobin | -/- | |
| | | | | Increase in serum IgM | 100/25 | |
| BALB/c mice | 5–8 | 4–8 weeks | 0, 12, 24 mg NIV/kg diet | Increase in serum IgA (4 weeks) | 24/12 | [36] |
| | | | | Increase in serum IgA (8 weeks) | 12/- | |

LOAEL, lowest observed adverse effect level; NOAEL, no-observed-adverse-effect level; bw, body weight; -, not mentioned.

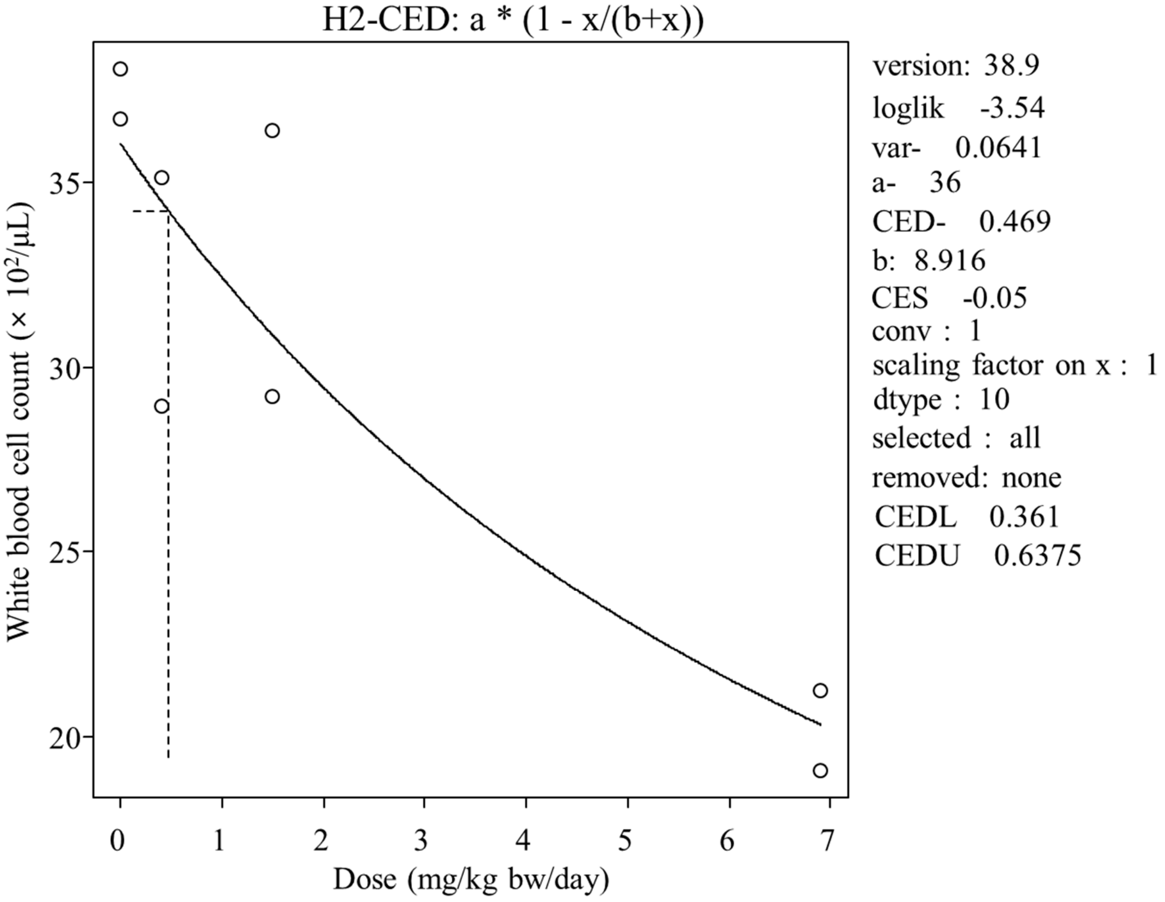


**Figure S1.** Dose–response modeling for white blood cell change with fitted Hill family model H2 of the 90 days subchronic study.
